# Plant-Derived Phenolic Acids Limit the Pathogenesis of *Salmonella* Typhimurium and Protect Intestinal Epithelial Cells during Their Interactions

**DOI:** 10.3390/molecules29061364

**Published:** 2024-03-19

**Authors:** Zabdiel Alvarado-Martinez, Zajeba Tabashsum, Arpita Aditya, Katherine Hshieh, Grace Suh, Matthew Wall, Aaron Scriba, George Sellers, Christa Canagarajah, Sarika Kapadia, Debabrata Biswas

**Affiliations:** 1 Biological Sciences Program-Molecular and Cellular Biology, University of Maryland, College Park, MD 20742, USA; zalvara1@terpmail.umd.edu (Z.A.-M.); ztabashs@terpmail.umd.edu (Z.T.); 2 Department of Animal and Avian Sciences, University of Maryland, College Park, MD 20742, USA; aaditya1@terpmail.umd.edu; 3 Department of Biology, University of Maryland, College Park, MD 20742, USA; khshieh@terpmail.umd.edu (K.H.); ssgrace737@gmail.com (G.S.); mjwall@terpmail.umd.edu (M.W.); aascriba@terpmail.umd.edu (A.S.); gsellers@terpmail.umd.edu (G.S.); ccanaga@terpmail.umd.edu (C.C.); sarikakapadia4@gmail.com (S.K.)

**Keywords:** gallic acid, protocatechuic acid, vanillic acid, *Salmonella* Typhimurium, INT-407, invasion, adhesion

## Abstract

The incidence of gastrointestinal illness attributable to *Salmonella enterica* serovar Typhimurium (ST) remains a concern for public health worldwide, as it can progress into systemic infections mediated by the type-three secretion system (T3SS), which allows for adherence and invasion to intestinal epithelial cells. The current study evaluates the ability of gallic acid (GA), protocatechuic acid (PA), and vanillic acid (VA) to impair the adhesion and invasion abilities of ST to a human epithelial (INT-407) cell monolayer while also assessing their cytotoxicity. GA, PA, and VA inhibited detectable ST growth at specific concentrations but showed cytotoxicity against INT-407 cells (>20% reduction in viability) after 3 h of treatments. Adjusting the pH of the solutions had a neutralizing effect on cytotoxicity, though it did reduce their antimicrobial potency. Adhesion of ST was reduced significantly when the cells were treated with 4.0 mg/mL of VA, whereas invasion was reduced in all treatments, with GA requiring the lowest concentration (0.5 mg/mL). Relative gene expression of virulence genes after treatment with GA showed downregulation in the T3SS regulator and effector *hilA* and *sipA*, respectively. These findings suggest further use of phenolic acids in reducing the activity of key virulence factors critical during ST infection.

## 1. Introduction

Non-typhoidal *Salmonella enterica* is a prominent pathogen that is estimated to be responsible for millions of cases around the world, many of which result in death, making it a major causative agent of gastroenteritis, with one of its main infectious routs being the consumption of contaminated food [1,2]. Despite the numerous measures taken to reduce its prevalence, non-typhoidal *S. enterica* also remains one of the major etiological agents of gastrointestinal disease worldwide, including in developed countries such as the USA, where it remains the main bacteria responsible for domestically acquired bacterial cases of illness, hospitalizations, deaths, and outbreaks [3,4]. *S. enterica* has over 2500 serovars, many of which are common etiological agents of disease and outbreaks in the USA [5]. However, *S. enterica* serovar Typhimurium (ST) remains one of the most clinically relevant serovars given its ubiquitous prevalence across the environment and various food categories, as well as for its broad antibiotic resistance pattern and well-adapted virulence factors that aid it during infection of its human hosts [6,7,8,9]. Though most cases of infection with ST are considered to be self-limiting and mostly manifest as a gastrointestinal disease, infections have been known to persist as they develop into systemic infections that can spread to bones, joints, and bloodstream, especially within vulnerable populations, such as children, the elderly, immunocompromised patients and people that cannot clear out the infection in time or are suffering from a secondary infection [10]. Various control measures have been proposed, including vaccination and the development of new-generation antibiotics; however, these have been met with limitations that currently lower their current applicability as proper preventive measures and as treatments, as they also require more research to be implemented safely and effectively [11]. On the other hand, extracts from plants have also demonstrated promising antimicrobial activity against ST and have the potential to be abundant sources of antimicrobial compounds that do not contribute to the development of antibiotic resistance while also not affecting host cells or the host probiotic and commensal bacterial microflora [12,13,14].

Purified plant-derived compounds commonly found in plant extracts have also been proposed as potential methods of control against ST, given their diversity and potential for multiple mechanisms of action that reduce the likelihood of antibiotic resistance while preserving and sometimes promoting the growth and integrity of pro-commensal bacteria in the gastrointestinal (GI) tract of the human host [15]. Plant extracts are known to contain thousands of bioactive molecules, many of which are polyphenolic compounds [16]. These phenolic compounds can be further categorized as flavonoids, tannins, stilbenes, lignans, or phenolic acids, with the latter accounting for one-third of the total phenolic compounds found in some plants [17]. Phenolic acids, in particular, have been reported to be bioactive, as they display antimicrobial potential against various species of bacteria in vitro while also being useful as food preservatives and antioxidants [17,18]. Previous studies have tested the efficacy of specifically gallic acid (GA), protocatechuic acid (PA), and vanillic acid (VA) against ST in vitro, with findings demonstrating these compounds to exert antimicrobial activity against the bacteria while also showing downregulation of important virulence genes associated with invasion of epithelial cells [19].

The capability to adhere to and later invade multiple cell types within the gastrointestinal (GI) tract of a human host, especially intestinal epithelial cells, is an important virulence factor of ST that creates further challenges for effective treatments [20]. These virulence factors serve as a survival mechanism that allows the bacteria to avoid natural shedding by the host, as well as neutralization by the immune system, while also allowing the pathogen to proliferate intracellularly and potentially spread systemically if it reaches deeper layers of tissues [21]. Recent studies have revealed numerous pathways that ST utilizes for the invasion of non-phagocytic epithelial cells, providing ST with numerous mechanisms of entry. However, one of the best-studied and well-described mechanisms of action remains the type 3 secretion system (T3SS), encoded in the *Salmonella* pathogenicity island 1 (SPI-1), which is highly conserved, especially in ST and has also been established as a crucial model for studying the host-pathogens interactions of ST within the context of infection both in vitro and in vivo [22,23]. In addition to the T3SS, ST uses other virulence like flagella-mediated motility to penetrate the layers of mucus and commensal bacteria that line the GI tract in order to reach the epithelial cells and initiate invasion [24]. For these reasons, in addition to testing compounds for their direct antimicrobial capability, researchers have also suggested testing the anti-virulence potential of novel compounds, as these could serve as additional targets while also helping in the amelioration of invasion [25].

Considering the importance of virulence and the previously studied potential of phenolic acids to inhibit ST and reduce relative expression of virulence genes, the purpose of this study is to test the efficacy of GA, PA, and VA for preventing adhesion and invasion of ST during the infection of epithelial cell lines. For these purposes, INT-407 cell lines were chosen as a model, considering their susceptibility to ST invasion [26]. GA, PA, and VA were tested for cytotoxicity against INT-407 and later used as treatments against ST infection, which was determined directly through calculating colony forming unit (CFU)/mL, in addition to measuring the relative expression of virulence genes associated with regulation of the T3SS system and other virulence factors.

## 2. Results

### 2.1. Evaluation of Cytotoxicity of Phenolic Acids in INT-407 Cells Using MTT Assay

**Figure 1:**
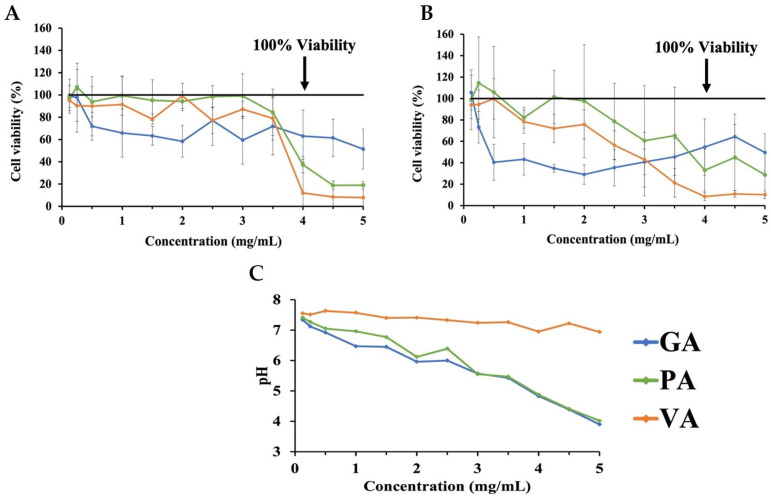
MTT assay assessing changes in cell viability as a result of cytotoxicity for INT-407 cell monolayer treated with increasing concentrations of GA, PA, and VA in DMEM supplemented with 10% FBS for 3 h (**A**) and 24 h (**B**), as well as the corresponding pH values for each phenolic acid concentration (**C**).

Increasing concentrations of DMEM with 10% FBS individually supplemented with either GA, PA, or VA were prepared and tested in an INT-407 cell monolayer to determine the potentially cytotoxic concentrations of the phenolic acids at various concentrations (Figure 1). MTT assays were performed on the cells treated with increasing concentrations of phenolic acids for 3 h (Figure 1A) and 24 h (Figure 1B), with measures being compared to an untreated control at the same time point. Concentration-dependent cytotoxicity at 3 h for GA resulted in a reduction of viability to 71.85% at 0.5 mg/mL, followed by a decrease to 58.32% at 2.0 mg/mL and 48.60% at 5.0 mg/mL. PA showed a decrease in cell viability to 37.39% at 4.0 mg/mL, while VA lowered cell viability to 78.32%, 77.13%, and 12.02% at 1.5 mg/mL, 2.5 mg/mL, and 4.0 mg/mL, respectively. After exposure to phenolic acids for 24 h, cells treated with GA had a decrease in cell viability to 73.32% at 0.25 mg/mL and were most cytotoxic at 2.0 mg/mL, which reduced viability to 29.07%. Treatment with PA over 24 h reduced viability to 78.66%, beginning at 2.5 mg/mL, and had a major decrease to 33.10% at 4.0 mg/mL. Cells treated with VA showed an initial decrease in viability to 78.35% at 1.0 mg/mL, with the most significant decrease having been detected at 4.0 mg/mL with a reduction to 8.50%. Measuring the natural pH of increasing concentrations of each phenolic acid combination in DMEM showed these to be inversely correlated, as increasing concentrations led to a decrease in the pH (Figure 1C). At a concentration of 0.0625 mg/mL, GA, PA, and VA had a pH of 7.34, 7.41, and 7.55, respectively, with these decreasing to 4.83, 4.88, and 6.98, respectively, at 4.0 mg/mL.

### 2.2. Determining Effects of pH on Cytotoxicity in INT-407 Cell Using MTT

**Figure 2:**
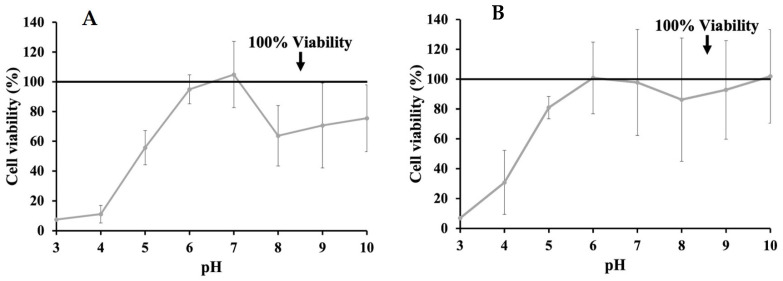
Effects of changing pH of DMEM supplemented with 10% FBS on cell viability evaluated through MTT assay after 3 h (**A**) and 24 h (**B**).

**Figure 3:**
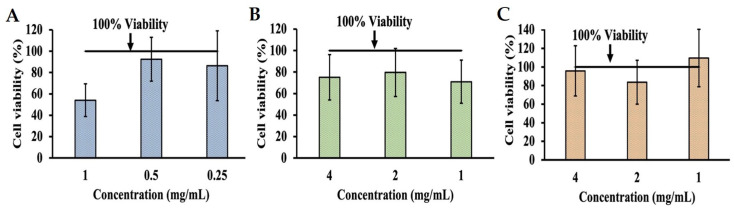
MTT assay assessing changes in viability of cell line as a result of cytotoxicity after adjusting DMEM with 10% FBS to a pH of 7 when also supplemented with GA (**A**), PA (**B**), and VA (**C**).

Additional MTT assays were performed on INT-407 cells incubated in DMEM with 10% FBS adjusted to a pH ranging from 3 to 10 in order to determine the effects of pH alone on cell viability by comparing these to unmodified DMEM (Figure 2). MTT assays for cells incubated with the pH-adjusted DMEM were performed at 3 h (Figure 2A) and 24 h (Figure 2B). When assessing changes in cell viability at 3 h, the most significant decrease in viability was to 7.00% and 30.77% at pH 3 and 4, respectively. However, viability improved as pH increased, with pH 6 and 10 showing an increase in proliferation to 100.82% and 101.86%, while pH 7 only had a reduction in viability to 97.79%. When extending incubation time to 24 h, viability at pH 3 and 4 remained the lowest at 7.51% and 11.13%, respectively, though it also improved as pH increased, with pH 6 showing a viability of 94.94%, while pH 7 had a proliferative effect on INT-407 cells increasing viability to 104.86%. In order to evaluate whether the cytotoxic effects of phenolic acids could be neutralized through pH modulation, concentrations of phenolic acids previously determined to be cytotoxic were adjusted to a pH of 7, with MTT assays being performed at 3 h (Figure 3). After adjusting these to pH 7, GA (Figure 3A) saw improvement in cytotoxicity at 1.0 mg/mL (54.11%) but remained below the 80% cutoff; however, at 0.5 mg/mL, there was an increase to 92.45% and 86.25% at 0.25 mg/mL. Adjusting PA (Figure 3B) had a similar effect at 4.0 mg/mL, increasing viability to 75.08%. However, viability decreased to 79.59% and 71.03% for 2.0 mg/mL and 1.0 mg/mL, respectively. Adjusting VA (Figure 3C) at 4.0 mg/mL had an improvement in viability exhibited by an increase to 95.83%, while 2.0 mg/mL showed a reduction to 83.65% and 1.0 mg/mL to 109.58%, all above the 80% cutoff established initially.

### 2.3. Antimicrobial Potency of Phenolic Acids in DMEM

**Figure 4:**
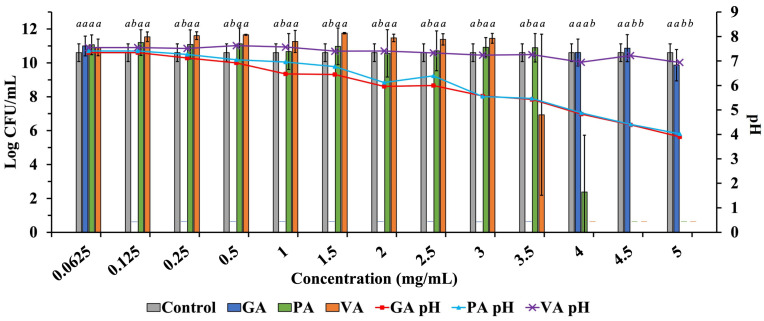
Testing increasing concentrations of GA, PA, and VA in DMEM supplemented with 10% FBS to determine the antimicrobial profile of phenolic acids when tested in DMEM, along with the corresponding pH at given concentrations. Statistically significant differences (*p* < 0.05) between treatments when compared to the untreated control at their respective time points were denoted with letters *a*-*b*. Differences between treatments were evaluated using ANOVA with a significance level set at *p* < 0.05.

Growth of ST was quantified by calculating Log CFU/mL in samples inoculated and incubated in only DMEM with 10% FBS or in DMEM with 10% FBS, individually supplemented with increasing concentrations of either GA, PA, or VA, and the growth pattern was compared to that of the untreated control to detect the antimicrobial activity of the phenolic acids in DMEM (Figure 4). Growth of ST in the untreated control was 10.60 Log CFU/mL. Supplementation with GA reduced ST to undetectable levels between the concentrations of 0.125 mg/mL and 3.5 mg/mL but showed growth at 4.0 mg/mL (7.96 Log CFU/mL), 4.5 mg/mL (10.87 Log CFU/mL) and 5.0 mg/mL (9.86 Log CFU/mL). PA reduced the bacterial load of ST to undetectable levels at 4.5 mg/mL and above, with the next significant reduction in ST being observed at 0.5 mg/mL (7.42 Log CFU/mL). VA was able to reduce ST to undetectable levels at 4.0 mg/mL and above, while bacterial load increased as concentration decreased, with 3.5 mg/mL showing the next most significant reduction of ST (4.62 Log CFU/mL).

Further tests were performed to determine the antimicrobial potential of increasing concentrations of phenolic acids (0.0625–5.0 mg/mL) in DMEM after adjusting the solution to a pH value ranging from 3 to 10, which was compared to an un-supplemented DMEM control also adjusted to specific pH values (Table 1). Adjusting pH changed the antimicrobial activity of phenolic acids in DMEM compared to their natural pH at their respective concentration, leading to either inactivation in previously determined lethal doses of the compounds or improved potency in previously inactive doses; however, changes in pH did not have an impact on growth when in DMEM alone, as ST growth was detected in all values within the pH range tested. In samples supplemented with GA, there was a loss of antimicrobial function at all pH values for 0.0625 mg/mL, while those for 0.125 mg/mL lost antimicrobial function between the range of pH of 4 and 7. Antimicrobial potency was regained at pH 7 for concentrations of 0.25 mg/mL and above but remained neutralized for all concentrations at pH 4. Similarly, PA showed inactivation at 0.0625 mg/mL for all pH ranges but was active at 0.125 mg/mL and above when the pH was 3. The pH range between 6 and 8 rendered PA ineffective regardless of concentration, though it regained antimicrobial activity at 1.0 mg/mL for pH 9 and 10, as well as at pH 4 when increasing the dose to 1.5 mg/mL. VA retained antimicrobial potency up to the concentration of 3.5 mg/mL, where it lost activity at pH 6 and above but was effective at 4.0 mg/mL regardless of the pH. At 2.5 mg/mL, VA lost total activity at every pH test. When adjusted to pH 7, only GA at 0.25 mg/mL and above, as well as VA at 4.5 mg/mL, were able to completely inhibit ST.

### 2.4. Changes in Host-Pathogen Interactions between ST and INT-407 after Treatment

**Figure 5:**
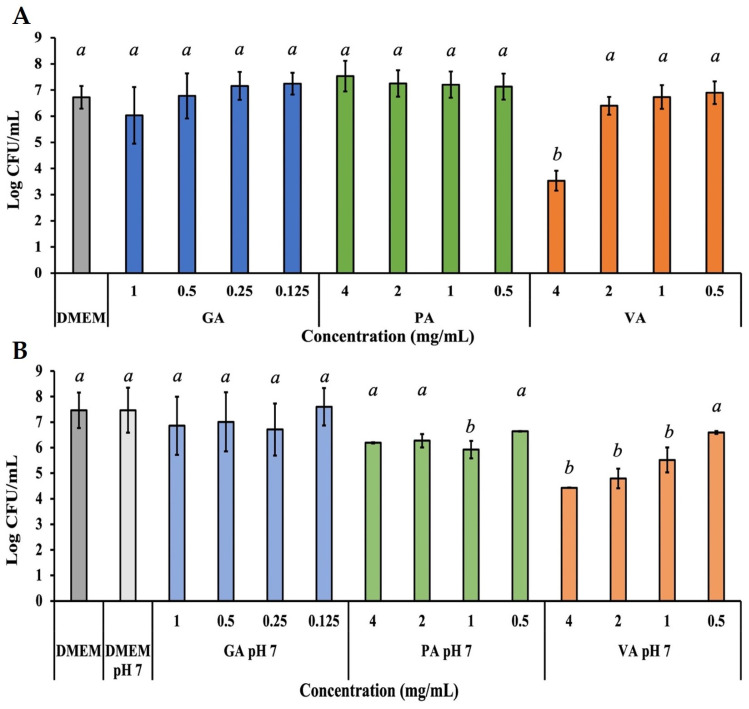
Bacterial quantification of ST adhered to INT-407 cell monolayer after 3 h of incubation with a treatment dose of either GA, PA, or VA in DMEM supplemented with 10% FBS at their normal pH (**A**) or adjusted to a pH of 7 (**B**). Statistically significant differences (*p* < 0.05) between treatments when compared to the untreated control at their respective time points were denoted with letters *a*-*b*. Differences between treatments were evaluated using ANOVA with a significance level set at *p* < 0.05.

The adhesion capability of ST to an INT-407 cell monolayer as a result of treatment with each phenolic acid was individually evaluated, considering the cytotoxicity of each compound, as well as their previously determined effective antimicrobial concentrations (Figure 5A). Unadhered bacteria suspended in the media were removed, and adhered ST were quantified from a cell monolayer after 0.1% Triton X-100 lysis, which releases the adhered bacteria and allows for calculating Log CFU/mL from a microdilution plating assay. Treatment with GA only showed a slight numerical decrease in adhered bacteria to 89.71% at 1.0 mg/mL, while PA showed no adverse effect on adhesion at any concentration. Exposure to VA at 4.0 mg/mL was the only treatment to show a statistically significant reduction (*p* < 0.05) of adhesion, shown in a reduction of 52.51%, as compared to the untreated control. Further assays were performed by adjusting the pH of the phenolic acids and DMEM solution (Figure 5B). The change in pH alone had no effect on the adhesive capability of ST. In the case of GA and PA, there was no significant change in adhesion, with the exception of PA at 1.0 mg/mL, which showed a significant (*p* < 0.05) decrease to 79.33%. VA showed a statistically significant (*p* < 0.05) reduction to 59.33%, 64.20%, and 73.92% at concentrations of 4, 2, and 1.0 mg/mL, respectively.

**Figure 6:**
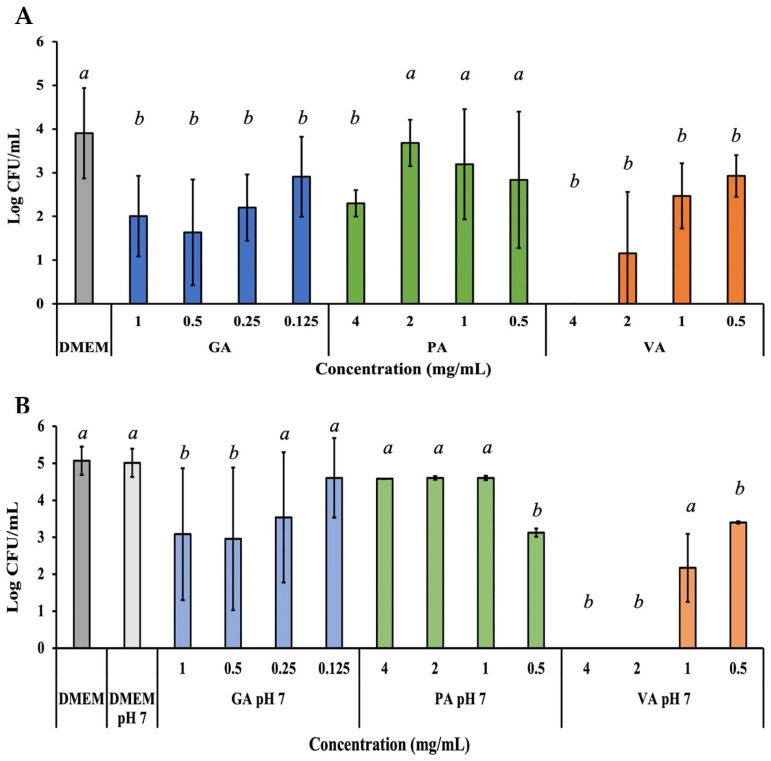
Bacterial quantification of intracellular ST that invaded the INT-407 cell monolayer after 3 h of incubation with a treatment dose of either GA, PA, or VA in DMEM supplemented with 10% FBS at their normal pH (**A**) or adjusted to a pH of 7 (**B**). Statistically significant differences (*p* < 0.05) between treatments when compared to the untreated control at their respective time points were denoted with letters *a*-*b*. Differences between treatments were evaluated using ANOVA with a significance level set at *p* < 0.05.

The invasive capability of ST to an INT-407 cells monolayer during treatment was evaluated in a similar way to adherence, with the addition of a gentamycin treatment step to eliminate all external ST, leaving only intracellular ST that was later collected by lysing the cell monolayer with a 0.1% Triton X-100 lysis, and quantified using the microdilution assay (Figure 6A). Treatment with GA showed a statistically significant (*p* < 0.05) reduction in invasion of ST at all the concentrations tested, with 0.5 mg/mL having the greatest reduction to 40.24%, followed by a reduction to 49.45% at 1.0 mg/mL. PA treatment was only able to significantly (*p* < 0.05) reduce ST to 56.66% at 4.0 mg/mL, with the remaining concentrations being only numerically lower. VA at 4.0 mg/mL had the greatest reduction, exhibited by inhibition of ST to non-detectable levels, though lower concentrations had the ability to also significantly (*p* < 0.05) reduce ST to 28.50%, 60.83% and 72.10% at concentrations of 2.0, 1.0, and 0.5 mg/mL, respectively. Further adjusting the pH of the phenolic acid and DMEM solutions lead to improved inhibitory activity for some compounds (Figure 6B). GA continued to show significant (*p* < 0.05) inhibitory capability at 0.5 mg/mL by reducing intracellular ST to 58.95%, followed by a reduction to 61.49% at 1.0 mg/mL (1.93 Log CFU/mL). PA showed a numerical decrease in invasion, but bacterial load at 0.5 mg/mL was significant (*p* < 0.05), as it was reduced to 62.36%. VA saw an extension in activity range, as in addition to 4.0 mg/mL, 2.0 mg/mL showed inhibition of ST to non-detectable levels, while lower concentrations like 1.0 mg/mL and 0.5 mg/mL remained active.

### 2.5. Relative Gene Expression of Virulence Genes and Inflammatory Cytokine Genes

**Figure 7:**
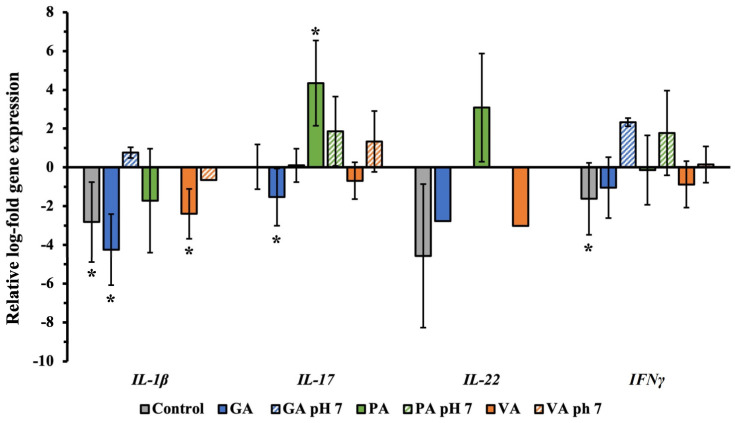
Relative log-fold expression of host genes associated with the production of inflammatory cytokines during ST infection, using *GAPDH* as the housekeeping gene and uninfected cells as control for normalization. A statistically significant (*p* < 0.05) difference in treatment as compared to the control was denoted by a star (*).

The total RNA was collected from INT-407 cell monolayer individually treated with each phenolic acid to evaluate changes in the relative expression of genes associated with inflammatory cytokine production using qRT-PCR (Figure 7). Average Cq values were recorded and normalized with *GAPDH* as the housekeeping genes, with an untreated sample serving as the control, while results were represented in Log form. Infected cells showed a significant (*p* < 0.05) downregulation of *IL-1β* to −2.82 Log, while there was a numerical decrease in *IL-22* and *IFNγ* to −4.57 and −1.63 Log, respectively. When treated with GA, there was a greater significant reduction in *IL-1β* and *IL-17* to −4.26 and −1.53 Log. Conversely, treatment with PA upregulated *IL-17* expression to 4.34 Log, while VA downregulated *IL-1β* to −2.40 Log. Changing the pH of GA to 7 showed a numerical upregulation of 0.76 Log for *IL-1β* and 2.33 Log in *IFNγ*, while pH adjusted pH had an upregulation of 1.86 Log and 1.78 Log for *IL-17* and *IFNγ*. For samples from pH-adjusted treatments, there was no expression of *IL-22*.

**Figure 8:**
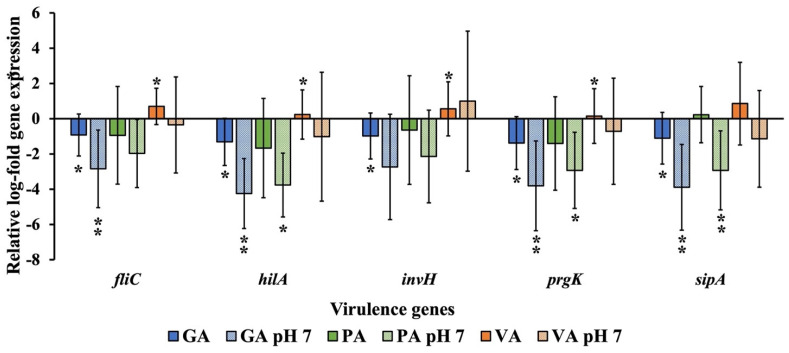
Relative log-fold expression of ST genes is responsible for regulating virulence, using the *16S-rRNA* gene as the housekeeping gene and an untreated control for normalization. A statistically significant difference in treatment as compared to the control was denoted by a star (*) (*p* < 0.05) or two stars (**) (*p* < 0.01).

Changes in the expression of ST virulence genes were also evaluated from total RNA extracted from cells and bacteria collected during the process of infection and later evaluated through the use of qRT-PCR and gene-specific primers for *fliC*, *hilA*, *invH*, *prgK*, and *sipA*, with *16S-rRNA* gene being used as the housekeeping gene, and an infected but untreated sample being used as the untreated control (Figure 8). Samples treated with GA all showed significant (*p* < 0.05) downregulation of the genes tested, most notably for *prgK* (1.38 Log) and *sipA* (1.11Log), though adjusting to pH 7 led to an even greater downregulation of all genes, especially *hilA* (4.25 Log), though *prgK* (3.81 Log) and *sipA* (3.89 Log) remained low. PA treatment led to downregulation of every gene, with the exception of *sipA* (0.23 Log), which showed to be slightly upregulated, though none were statistically significant. Adjusting PA to pH 7 exhibited a greater downregulatory effect as there was a significant (*p* < 0.05) reduction of *hilA* (3.76 Log), *prgK* (2.93 Log) and *sipA* (2.93 Log). VA treatment exhibited a significant (*p* < 0.05) upregulation of most genes, especially *fliC* (0.70 Log), followed by *invH* (0.56 Log). Adjusting the pH of VA led to a downregulation of all genes except *invH* (1.00 Log). Though none were statistically significant, *sipA* (1.14 Log) and *invH* (1.02 Log) had the most notable numerical difference.

## 3. Discussion

Plants remain significant contributors to the discovery of many bioactive compounds that could be used for their application in controlling many pathogenic bacteria, especially ST. The phenolic compounds within plants have been identified as being notable candidates given their abundance and diversity, which are key factors that contribute to these being able to be used as antimicrobials without contributing to the increasing trends in antibiotic resistance and remain effective for longer periods of time [15]. These compounds have also garnered attention for their antioxidant and anti-inflammatory properties when tested in various types of eukaryotic host cell lines [27,28,29]. Phenolic acids specifically have been studied in the past for their antimicrobial activity against multiple pathogenic bacteria, their long use as food preservatives, and their bioactive byproducts [30,31,32]. Their use against ST specifically has been assessed in vitro in the past, as well as their efficacy when used in conjunction with other antimicrobials to enact a synergistic effect [19,33].

Direct inhibition of bacterial proliferation through growth inhibition remains one of the primary methods for control of ST, though recent studies have also been drawing attention to the importance of preventing colonization of host tissues, as well as targeting other virulence factors that the bacteria use to persist in infection [34]. Using novel compounds that can prevent adhesion and/or invasion of ST to epithelial tissue has been proposed as a potential target for alleviating the disease caused by ST while also preventing further progression and complications of the infection, as it will allow the host to shed the bacteria [35]. Despite the GI tract having numerous innate defense mechanisms, including digestive fluids, multiple layers of mucous and commensal/probiotic bacteria, as well as the immune system cells and cytokines, ST is also equipped to either bypass these adverse conditions or even use them to trigger invasion, therefore requiring further intervention to eliminate the pathogen [36]. One proposed invasion model suggests ST can overgrow in the GI tract and even outcompete the local microbial flora in cases where the intestine is inflamed, which leads to the release of reactive oxygen species (ROS), as well as the fermentation of other products like H_2_S, which provides ST with an additional nutrient source [37].

By testing GA, PA, and VA against ST in an INT-407 cell model, this study was able to assess the potential applicability of these compounds for the purposes of reducing the progression of ST infection in the GI tract. As mentioned before, though the GI tract is a complex ecosystem with many variables that can affect the efficacy of an antimicrobial product, cell monolayers have long been used as models for studying host-pathogen interactions between bacteria and eukaryotic cells [38]. One of the factors that affects the potency of phenolic acids is pH, which has been shown in the past to either increase their antimicrobial effect or neutralize them [39]. The initial antimicrobial assay performed in DMEM with 10% FBS showed that inhibitory potential increased with concentration in the case of PA and VA, though they required high doses in order to inhibit ST to undetectable levels. GA showed an uncommon inhibitory pattern where, despite the higher concentrations of GA, the corresponding pH at these could have a neutralizing effect on bacterial inhibition. Certain pH values neutralized GA, which could explain the loss of activity at higher concentrations. Changes in pH had a similar effect on PA and VA, as their activity varied across concentration and pH ranges. This effect of pH could be explained by changes in compound stability, as well as how these conditions can change the protonated state of the compounds, leading to either increased activity or a reduction based on how they will be able to interact with the bacteria [40].

The efficacy at specific pH was of importance for optimizing compound activity but was shown to be important when considering experiments involving the use of INT-407 cells. Epithelial cells were shown to be susceptible to changes in the pH of the media they were incubated in, resulting in a decrease in cell viability. The optimal pH for cell survival was determined to be 7. When testing for the cytotoxicity of each compound on INT-407, there was a noticeable decrease in viability as concentrations of each compound increased, though it is important to note that some can lose potency over time as they become susceptible to oxidization. The cutoff for cytotoxicity that was chosen was 80%, meaning a reduction in viability of >20%, which meant the compound induced cell death and could be potentially cytotoxic at those concentrations [41,42]. The drop in viability for GA was recorded at lower concentrations than for PA and VA, which led to the use of lower concentrations in general for GA to avoid cytotoxicity, in addition to previously showing activity at these lower concentrations. PA and VA were overall less cytotoxic, requiring higher concentrations to significantly reduce cell viability. Adjusting the pH of the final phenolic acid and DMEM solution to 7 had a reduction in the cytotoxicity for previously cytotoxic concentrations of some of the compounds. Actively adjusting the pH to 7 in GA showed an improvement for the 0.5 mg/mL solution despite the unadjusted pH for this concentration being similar to 7. This difference could be attributed to the pH adjustment process, which requires the use of NaOH and HCl, depending on the case.

Adhesion and invasion were used as the markers for assessing the host-pathogen interactions between ST and INT-407 during an infection period and treatment with each phenolic acid. The MOI that was chosen for these assays has been reported to represent more closely, and it was found in an in vivo condition [43,44]. Adhesion is an important step for initiating infection that will progress to invasion of the host cells, which in turn is mediated by the T3SS [45]. Treatment with GA or PA did not show a significant reduction in the adhesive capability, while VA did have activity at the higher doses, though this could also be the result of changes in the host cell, given this concentration was found to have cytotoxic effects. Adjusting the pH to 7 did not improve GA activity, though it did enhance that of PA and VA. Though adjusting to pH 7 in previous experiments had a neutralizing effect on the antimicrobial potential against ST growth, other researchers have reported similar scenarios in which specific natural compounds do not affect ST growth but still display activity for inhibiting specific functions within the bacteria, such as inhibiting the T3SS [46]. When measuring invasion, the amount of intracellular ST detected was found to be the lowest when treated with GA, which was further improved after adjusting to pH 7. Though the other compounds showed an ability to inhibit invasion, GA achieved the greatest reduction with the lowest concentration. VA had the most significant reduction at higher concentrations, exhibited by a reduction in ST to undetectable levels, which was later expanded after adjusting to pH 7. Previous research has shown the importance of pH as a key signal for ST to initiate the invasion of a host cell [47], but in the current study, a similar adhesion and invasion pattern for both 10% FBS-supplemented DMEM and pH 7 DMEM suggests no adverse effect exerted by pH alone, however, when paired with a specific concentration of phenolic acid, there could be an improvement in activity, whether mediated by the corresponding state of the compound or by the response of either ST or INT-407.

Quantifying changes in relative expression for INT-407 cell genes associated with the production of inflammatory cytokines gave information pertaining to the potential anti-inflammatory activity of each phenolic acid, which could have significant effects on the progression of ST infections. In an infection model, the production of cytokines like *IFNγ* has been found to lead to better outcomes in terms of infection progression [48]. Though not statistically significant, adjusting the pH of GA showed an upregulation of the *IFNγ* gene compared to its normal effect, which had a reduction. However, it is important to note that the values for *IFNγ* expression were higher than when left untreated. Similarly, IL-17 has been found to have a critical role in the early stages of infection, with studies showing its suppression to lead to greater epithelial damage [49]. The current study showed GA to downregulate *IL-17* genes, with adjusting to pH 7 leading to an increase showing slight upregulation, while PA had a significant increase in upregulation. In the case of IL-22, this cytokine has also been associated with a protective function during ST infection but has recently been shown to aggravate the infection in some cases [50]. This variation in outcomes makes it uncertain what the optimal outcome would be when using phenolic acids to modulate cytokine expression. The current study also showed no detection of expression of this gene when adjusting the pH of the treatments to pH 7, suggesting that transcription might have been suppressed due to this adjustment or the presence of the bacteria, as has been seen with other pathogens capable of suppressing transcription [51]. In the case of *IL-1β*, which is associated with inducing a pyroptotic response when in contact with bacterial lipopolysaccharides [52,53], treatment with GA significantly lowered the levels of expression, though adjusting to pH 7 had a slight numerical increase.

Relative expression of ST virulence genes during infection gave insight into the mechanism that might be behind the inhibition of invasion. Among the genes tested that are directly associated with invasion, *hilA* is a major transcriptional regulator, *invH* is mainly responsible for protein translocation, *prgK* is a structure protein that serves as the base of the T3SS, while *sipA* has been shown to be critical in the manipulation of actin filaments, mediating bacterial entry [54,55,56,57]. On the other hand, though *fliC* is not directly associated with invasion, it is still considered an important virulence factor since it mediates motility that can help bypass host defenses [58]. GA had a significant downregulatory effect, which was further reduced by adjusting to pH 7. In the case of VA, upregulation of the virulence genes was tested, suggesting a difference in mechanism of action, considering that VA was able to reduce invasion.

## 4. Materials and Methods

### 4.1. Bacterial Strain and Growth Conditions

*Salmonella enterica* serovar Typhimurium (ATCC 14028) (ST) was used for this study as the infectious agent being studied. Luria-Bertani (LB) agar (Becton, Dickinson and Co., Franklin Lakes, NJ, USA) was used as the medium on which ST was grown at 37 °C under aerobic conditions (Thermo Fisher Scientific Inc., Waltham, MA, USA). ST was cultured on LB agar using streaking from a glycerol stock and incubated overnight to obtain individual colonies, which were used later to prepare bacterial suspensions for future experiments.

### 4.2. Cell Line and Growth Conditions

Human intestinal epithelial cells (INT-407) (ATCC CCL-6) were cultured in Dulbecco’s Modified Eagle Medium (DMEM) (Corning Cellgro, Manassas, VA, USA) supplemented with 10% fetal bovine serum (FBS) (Atlanta Biologicals^®^, Lawrenceville, GA, USA) and 50 μg/mL gentamycin (IBI Scientific, Dubuque, IA, USA). All DMEM used in this study was prepared with 10% FBS unless stated otherwise for a specific assay. Cells were incubated at 37 °C in 5% CO_2_ and 95% humidity to achieve a 90% confluent monolayer (Thermo Fisher Scientific Inc., USA). Confluent cells were washed with PBS, detached from the cell culture flask using 0.25% trypsin (Gibco, Norristown, PA, USA), centrifuged, and resuspended for seeding into either a 96- or 24-well plate with fresh DMEM, free of gentamycin, and incubated under the same conditions until 90% of the monolayer is confluent before using for experiments.

### 4.3. Compounds and Stock Solution Preparation

The phenolic acids that were tested in this study were purchased from commercial vendors in powdered form for the preparation of working stocks. Gallic acid (GA) (Acros Organics, Doral, FL, USA) and protocatechuic acid (PA) (Sigma-Aldrich, St. Louis, MO, USA) by dissolving in sterile deionized water, while vanillic acid (VA) (Alfa Aesar, Ward Hill, MA, USA) was prepared by dissolving in 30% ethanol (Pharmco-Aaper, Brookfield, CT, USA). Aqueous solutions of sodium hydroxide (NaOH) and hydrochloric acid (HCl) were both prepared to a molarity of 10 M and used for adjusting the pH values of other solutions in this study. GA, PA, and VA were prepared to contain a concentration of 10 mg/mL. Phosphate buffer saline (PBS) was prepared to a pH of 7.2. Working stock solution of 3-(4,5-dimethylthiazol-2-yl)-2,5-diphenyltetrazolium bromide tetrazolium (MTT) (USA) was prepared by dissolving MTT powder in 1 × PBS to a concentration of 5.0 mg/mL.

### 4.4. Phenolic Acid Antimicrobial Potential within DMEM and at Alternate pH Ranges

The antimicrobial potential of GA, PA, and VA was tested in DMEM with 10% FBS by determining the minimum inhibitory concentration (MIC) of each compound using the Clinical and Laboratory Standards Institute (CLSI) M100 guidelines [59]. Further analysis to determine the minimum bactericidal concentration (MBC) and concentration at which there was no detectable ST growth were performed through microplate dilution assay, as performed previously, with some modification [60]. Briefly, an overnight culture of ST from an LB agar plate was used to prepare a bacterial suspension in PBS, which was adjusted to an optical density (OD_600_) of 0.1, which equals 10^8^ colony-forming units (CFU)/mL. This bacterial suspension was diluted further for subsequent assays. Increasing concentrations of GA, PA, and VA were prepared, ranging from 0.0625 to 5.0 mg/mL in DMEM, and inoculated with ST to a final bacterial load of 10^4^ CFU/mL, in addition to an untreated control. These were later incubated at 37 °C and 5% CO_2_ for 24 h. After incubation, concentrations exhibiting no visibly detectable growth were determined to be the MIC and confirmed by subculturing and re-inoculating in a new LB agar plate, in addition to having 20 µL aliquots collected for further microplate dilution assay.

To determine changes in the antimicrobial efficacy of phenolic acid under various pH ranges within DMEM, increasing concentrations of phenolic acids within DMEM were adjusted to a pH between 3 and 10 and tested for inhibition of ST, as has been described previously with some modifications [39]. Briefly, aliquots of DMEM were either independently supplemented with increasing concentrations of GA, PA, and VA ranging from 0.0625 to 5.0 mg/mL or left untreated as the control lacking phenolic acids. In addition to testing supplementation with increasing concentrations of phenolic acids, these were tested after adjusting to a pH ranging from 3 to 10 using HCl or NaOH and measured using a pH probe (Metler Toledo, Columbus, OH, USA). Samples were inoculated with ST to achieve a final volume of 10^4^ CFU/mL and incubated for 24 h at 37 °C with 5% CO_2_. Growth of ST was determined visually, and the absence of growth was confirmed by subculturing from the sample to a new LB agar plate to allow for re-growth. Growth was interpreted as a decrease or loss of antimicrobial potency.

### 4.5. MTT Assay for Evaluating Cytotoxicity and Cell Viability

Cytotoxic effects of GA, PA, and VA against INT-407 cells were evaluated using an MTT reduction assay, as has been described in the past, with some modifications [61,62]. Briefly, INT-407 cells were seeded into 96-well plates and grown under 5% CO_2_ at 37 °C until a 90% confluent monolayer was formed. Confluent INT-407 cells were individually treated and incubated with increasing concentrations of either GA, PA, or VA in DMEM with 10%FBS, while the untreated control contained only DMEM with 10% FBS. Additional cytotoxicity tests were performed to determine the optimal pH for INT-407 cell viability and proliferation by adjusting DMEM with 10% FBS to a pH between 3 to 10, in addition to testing changes in cytotoxicity of GA, PA, and VA at the pH that supports cell line survivability. After incubation of the designated treatment testing condition, the media and treatment for each well were carefully aspirated, followed by an additional rinse with 100 µL of PBS once. Once PBS was removed, 100 µL MTT incubation media (1:10 MTT reagent to DMEM without FBS) was added to every well, and the plate was incubated for 2 h under 5% CO_2_ at 37 °C to allow for the formation of formazan crystals. MTT incubation media was carefully removed, leaving undissolved formazan crystals intact, which were later solubilized with 200 µL of dimethylsulfoxide (DMSO) and incubated for 15 min with agitation. After final incubation with DMSO, optical density (OD) was read at 550 nm and recorded for calculating cell survival. This assay was performed 3 h and 24 h after treatment with phenolic acids, with the former being the timepoint that was also chosen for adhesion and invasion experiments, while the latter being chosen to understand the long-term effects of exposure to the compounds and has a better understanding of their tolerance. INT-407 cells experienced adverse cytotoxic effects whenever cell viability was reduced by >20%, meaning cell viability must remain at 80% or above for the compound and corresponding concentration to be considered non-cytotoxic [63].

### 4.6. Adhesion and Invasion Assay

Impairment of ST adhesion and invasion to INT-407 after treatment with phenolic compounds was evaluated as described before with modifications [64]. Briefly, a 90% confluent monolayer of INT-407 (~5 Log cell/mL) was used to seed a new 24-well plate. Once the seeded cells reached a 90% confluence, they were ready to be infected with ST. A bacterial suspension of ST was prepared by resuspending colonies from an overnight culture in PBS and adjusted to an OD_600_ of 0.1, which was used to inoculate the 24-well plates with INT-407 monolayer in either DMEM with 10% FBS or DMEM with 10% FBS supplemented with experimental doses of GA, PA, and VA, as well as GA, PA and VA neutralized to pH 7. A final multiplicity of infection (MOI) of 10 (1:10 ratio of bacteria per cell) was used as the concentration for infection. After inoculation of ST, 24-well plates were incubated for 4 h, under 37 °C and 5% CO_2_, to allow time for cell-bacteria interaction. As defined previously for both adhesion and invasion assays, prior to plating, INT-407 cell monolayers were washed three times with PBS, eliminating free unadhered bacteria from the media, with wells for invasion assay being further treated with 250 μg/mL of gentamycin for 1 h, eliminating non-invasive bacteria. For both adhesion and invasion assays, cell monolayers were detached and lysed using 0.1% Triton X-100, with the resulting suspension being serially diluted, plated on LB agar, and incubated at 37 °C to later determine Log CFU/mL of adhered and invasive bacteria.

### 4.7. Assessing Relative Expression in INT-407 Cells and ST

Concentrations of GA, PA, and VA that were previously determined to be effective in reducing adhesion and/or invasion during ST infection and that did not exert deleterious cytotoxic effects were further used to treat both ST-infected and uninfected monolayers of INT-407 cells to later assess changes in relative gene expression for both bacteria and cell line. After the 4 h treatment period, the entire contents of the well were collected for RNA extraction, including supernatant with unadhered bacteria and detached cells, as well as the cell monolayer with or without bacteria, which was detached using 0.1% Triton X-100. Total RNA was extracted from the samples utilizing a TRIzol (TRI Reagent^®^) (Molecular Research Center Inc., Cincinnati, OH, USA) extraction protocol, as described previously, with some modifications [65]. Briefly, RNA from infected cell lines and uninfected ones were extracted separately, using the same protocol, which first involved centrifuging samples at 14,000× *g* for 10 min to collect pellet containing either INT-407 cells or INT-407 with ST, which was later resuspended in ice-cold 1 × PBS as the first wash step. The centrifuge step was repeated, and the pellet was resuspended in TRI Reagent^®^ (Molecular Research Center Inc., USA) to homogenize it and release total RNA from the sample, which was subsequently isolated. After extraction, total RNA concentrations were measured using a NanoDrop spectrophotometer (Thermo Fisher Scientific Inc., USA) and standardized before using them as templates for cDNA synthesis with the High-Capacity cDNA Reverse Transcription Kit (Applied Biosystems, San Francisco, CA, USA) following the instructions outlined by the manufacturer. Synthesis of cDNA was conducted using a thermocycler protocol set to incubate for 25 °C for 10 m, 37 °C for 120 m, and 85 °C for 5 m.

### 4.8. Measuring Gene Expression through qRT-PCR Assay

Changes in the relative expression of genes associated with inflammatory response in INT-407 cells, as well as for virulence genes in ST, were assessed using qRT-PCR with primers targeting genes associated with these functions. Custom primers were designed to target conserved regions of each respective gene being evaluated, with genes related to inflammatory response in INT-407 being compared to a GAPDH housekeeping gene (Table 2) [66,67,68], while genes related to virulence and invasion regulation in ST were compared to a 16S-rRNA housekeeping gene (Table 3) [69]. Reactions for the qRT-PCR were carried out using the PerfeCTa SYBR Green Fast Mix following the guidelines in the manufacture protocol (Quanta Bio, Beverly, MA, USA), with the reads and quantification being performed in the Eco Real-Time PCR system (Illumina, San Diego, CA, USA). The thermal profile for the qRT-PCR protocol was set to have 30 s at 95 °C, followed by 40 cycles of 5 s at 95 °C, 15 s at 55 °C and 10 s at 72 °C. The average Cq values reported by the qRT-PCR were recorded and later used to calculate the RQ value through normalization with the respective housekeeping gene and untreated control of the assay being performed. Values were reported and visualized as Log functions of the RQ values.

### 4.9. Statistical Analysis

All experiments were performed with biological triplicates. Student *t*-test was used to determine statistically significant (*p* < 0.05) changes between individually treated groups with the untreated control, as well as evaluated significant differences between treatment groups using ANOVA.

## 5. Conclusions

The use case of plant-derived antimicrobial compounds has been expanding, considering their activity. The current study has evaluated the applicability of GA, PA, and VA for inhibiting key virulence factors in ST, like adhesion and invasion, while also considering the effects that these treatments can have on the host cells. Results showed the potential that some of these compounds have for reducing invasion, particularly providing insight into future optimization steps that can be taken in order to enhance the efficacy of these compounds while also reducing detriment to the host cells.

## Figures and Tables

**Table 1:** Antimicrobial potential of increasing concentrations of phenolic acids in DMEM with adjusted pH.

pH		Concentration (mg/mL)																																						
	DMEM	Gallic Acid													Protocatechuic Acid													Vanillic Acid												
	0	0.06	0.12	0.25	0.5	1	1.5	2	2.5	3	3.5	4	4.5	5	0.06	0.12	0.25	0.5	1	1.5	2	2.5	3	3.5	4	4.5	5	0.06	0.12	0.25	0.5	1	1.5	2	2.5	3	3.5	4	4.5	5
3	+	+	-	-	-	-	-	-	-	-	-	-	-	-	+	-	-	-	-	-	-	-	-	-	-	-	-	+	+	+	+	+	+	+	-	-	-	-	-	-
4	+	+	+	+	+	+	+	+	+	+	+	+	+	+	+	+	+	+	+	-	-	-	-	-	-	-	-	+	+	+	+	+	+	+	-	-	-	-	-	-
5	+	+	+	+	+	+	+	+	+	+	+	+	+	+	+	+	+	+	+	+	+	-	-	-	-	-	-	+	+	+	+	+	+	+	-	-	-	-	-	-
6	+	+	+	+	+	+	-	-	-	-	-	-	-	-	+	+	+	+	+	+	+	+	+	+	+	+	+	+	+	+	+	+	+	+	+	+	+	+	-	-
7	+	+	+	-	-	-	-	-	-	-	-	-	-	-	+	+	+	+	+	+	+	+	+	+	+	+	+	+	+	+	+	+	+	+	+	+	+	+	-	+
8	+	+	-	-	-	-	-	-	-	-	-	-	-	-	+	+	+	+	+	-	-	-	+	+	+	+	+	+	+	+	+	+	+	+	+	+	+	+	-	-
9	+	+	-	-	-	-	-	-	-	-	-	-	-	-	+	+	+	+	-	-	-	-	+	-	-	-	-	+	+	+	+	+	+	+	+	+	+	-	-	-
10	+	+	-	-	-	-	-	-	-	-	-	-	-	-	+	+	+	+	-	-	-	-	+	-	-	-	-	+	+	+	+	+	+	+	+	+	+	-	-	-

In addition to visible detection, ST growth (+) or complete inhibition (-) was confirmed by spot platting aliquots taken from gallic acid, protocatechuic acid, and vanillic acid solutions in DMEM with 10% FBS, as well as the untreated control, which were later plated in fresh LB agar, incubated overnight and re-assessed for confirming growth.

**Table 2:** Primers used in quantifying gene expression of INT-407 cells.

Gene	Cytokine	Primer Sequence (5′-3′)
*GAPDH*	dehydrogenase	F: GGTGGTGCTAAGCGTGTTAT
		R: ACCTCTGTCATCTCTCCACA
*IFNγ*	interferon gamma	F: GTGAAGAAGGTGAAAGATATCATGGA
		R: GCTTTGCGCTGGATTCTCA
*IL-17*	neutrophil activation	F: GCAGATGACGGTACATCCAA
		R: CCAGATCAGGCTGTGCTTTA
*IL-1β*	induces pyroptosis	F: GCCATGGACAAGCTGAGGAAG
		R: GTGCTGATGTACCAGTTGGG
*IL-22*	host defense at mucosa	F: CTCCGATCCCTTATTCTCCTC
		R: AAGCGGTTGTGGTCCTCAT

**Table 3:** Primers used in quantifying gene expression of ST.

Gene	Protein	Primer Sequence (5′-3′)
*16S-rRNA*	16S ribosomal RNA protein	F: GTAGTACGATGGCGAAACTGC
		R: CTTCTCGACCCGAGGGACTT
*hilA*	SPI-1 transcriptional regulator	F: AATGGTCACAGGCTGAGGTG
		R: ACATCGTCGCGACTTGTGAA
*fliC*	flagellum subunit	F: GCAGATGACGGTACATCCAA
		R: CCAGATCAGGCTGTGCTTTA
*invH*	adherence and invasion	F: GGTGCCCCTCCCTTCCT
		R: TGCGTTGGCCAGTTGCT
*sipA*	actin binding protein	F: CGTCTTCGCCTCAGGAGAAT
		R: TGCCGGGCTCTTTCGTT
*prgK*	base structure formation of T3SS	F: GGGTGGAAATAGCGCAGATG
		R: TCAGCTCGCGGAGACGATA

## Data Availability

The data presented in this study are available in article.
